# Would endoscopic surgery be the gold standard for stapes surgery in the future? A systematic review and meta-analysis

**DOI:** 10.1007/s00405-020-06132-2

**Published:** 2020-07-09

**Authors:** Lucheng Fang, Jiayuan Xu, Wen Wang, Yideng Huang

**Affiliations:** 1 First Affiliated Hospital of Wenzhou Medical University, Wenzhou City, Zhejiang Province China; 2 Wenzhou Medical University, Wenzhou City, Zhejiang Province China

**Keywords:** Endoscopic, Microscopic, Otosclerosis, Congenital stapedial fixation, Stapes surgery

## Abstract

**Objective:**

This meta-analysis is aimed to review and analyze all available data of intraoperative and postoperative results of endoscopic and microscopic stapes surgery.

**Methods:**

According to the PRISMA statements checklist, this systematic review and meta-analysis were designed. Data were extracted from public databases, such as PubMed, Cochrane, Web of Science, and more. The quality of studies was evaluated using the MINORS scale. Odds ratios (ORs) and 95% CIs were estimated for binary outcome data, while the mean differences and 95% CIs were estimated for continuous data. *I*^2^ and *χ*^2^ tests were used to quantify statistical heterogeneity. If more than ten studies were included in each analysis, funnel plot would be performed to analysis publication bias.

**Results:**

Twelve studies with 620 patients were included in this meta-analysis. Primary outcomes collected in this meta-analysis included average postoperative auditory gain (APAG), postoperative air–bone gap (ABG), the rate of chorda tympani handling and bone curettage, which all showed a statistically significant difference in favor of endoscopy. While only secondary outcomes about postoperative pain and dysgeusia demonstrated a significantly reduced incidence. Furthermore, there was not any statistically significant difference on postoperative dizziness and average operative time between endoscopy and microscopy.

**Conclusion:**

Although there is a need for high-quality pooled data in the future, a consistently superior effect of the endoscopic group was still shown in terms of total effectiveness, when compared to the microscopic group. We have reasons to support the application of endoscopy in stapes surgery. The future of ESS, we believe, is blazing bright.

## Introduction

Traditionally, microscopic surgery is the best choice to perform stapes surgery (SS), since Shea first described modern stapedectomy with a teflon implant in 1956 [1–3]. Although microscopic stapes surgery (MSS) nowadays is quite successful, some limitations still certainly exist. Primarily, the visualization of the tympanic membrane and middle-ear structures is limited, especially for patients who have narrow segment of the external auditory canal, because of the perennial problem of the tunnel vision which often contributes to the requirement of an external incision [1, 4, 5]. Consequently, endoscopic stapes surgery (ESS) is gaining growing attention all over the world. The main reason for this booming interest is the patients’ wish for minimal invasive surgery to avoid a painful incision. What’s more, advantages of ESS, such as providing better surgical field exposure with panoramic vision to hidden recesses, as well as complex structures, and reduced bone curettage, also satisfy the demand of surgeons. Additionally, the endoscope could lightly pass a narrow isthmus in the transcanal approach, resulting in the enhanced visualization of the ossicular chain and oval window clear [6–9]. Nevertheless, the endoscope is not perfect. The lack of depth perception because of two-dimensional vision, the operation of one hand, and the relatively long learning curve all make many otologists unwilling to switch to endoscopic technology [9–11]. Despite the awkward difficulty of endoscopic one-hand surgery, almost all of the surgical steps, theoretically speaking, involved in the stapes surgery procedures were practicable by endoscopy.

The SS is mainly applied to otosclerosis and congenital stape footplate fixation (CSFF), frequently presenting with an intact tympanic membrane and acquired conductive hearing loss, and stapedotomy has been widely regarded as the most efficient surgical modality for treatment [1, 3, 12]. Some previous clinical studies have indicated that an endoscopic approach, when compared with a traditional microscopic approach, has almost, even completely, similar hearing outcomes, while both rates of chorda tympani nerve(CTN) transection, as a matter of fact, and scutum removal are decreased likewise [2, 3, 7, 8, 13]. However, recognizing that individual studies probably cannot provide sufficient evidence on their own to affect clinical practice, we sought to objectively assess the difference between ESS and MSS. Therefore, this new systematic review and meta-analysis was reported to search and analysis all clinical data from available controlled studies, so that the similarities and differences between ESS and MSS could be compared in various aspects.

## Methods

### Study design and inclusion criteria

According to the PRISMA statements checklist [14], this systematic review and meta-analysis were performed.

Meticulously, we defined inclusion criteria before initiating the data search to ensure that all eligible studies are correctly identified. The study selection procedures were performed independently by two reviewers (X.J.Y. and W.W.) and disagreements were also resolved by consensus. The PICO (population, intervention, comparison, and outcome) framework applied as the selection criteria were as follows: (1) the population included patients with otosclerosis or congenital stapedial fixation. (2) Only controlled studies comparing ESS and MSS were analyzed. Case reports, case series, commentaries, conference abstracts, and review articles were not considered. (3) Postoperative outcomes, such as the average postoperative air–bone gap (ABG), the manipulation of CTN, postoperative dizziness and so on, were documented sufficiently [15].

### Literature search strategy

A literature search was performed in the PubMed, Cochrane, Web of Science, ClinicalTrials and EMBASE from inception up to January 1, 2020. We applied the following free-text terms to searching in the PubMed database: (otoscleros* OR otospongios* OR “congenital stapedial fixation” OR “congenital stapes fixation”) AND (stapedotom* OR stapedectom* OR “stapes surgery”) AND (endoscop* OR microscop*). In addition, we modified this search strategy to apply to search rules of other databases. Besides, we independent reviewers, to identify additional suitable studies, cross-checked both the reference lists of the available studies and relevant review articles.

### Data extraction

Two reviewers (X.J.Y. and W.W.) screened both the title and abstract, independently and blindly, of studies collected. For enhancing sensitivity of analysis, only when both reviewers excluded the record at the title and abstract screening level, could this record be definitely removed. A full-text analysis of the selected articles, subsequently, was performed by both reviewers. It was, if necessary, a third reviewer (F.L.C.) that resolved any divergence between the two reviewers in the selection and evaluation procedures. The following data from each selected study were extracted: characteristics (lead author, year of publication, country, study design, the amount of patients, study population, mean age, and mean length of follow-up) and outcomes data. Two independent reviewers assessed risk for bias according to the PRISMA recommendations and the Cochrane tool for assessing risk for bias [14, 16]. Subsequently, we made the qualitative and quantitative analyses of all data from the involved studies.

### Statistical analysis

We assessed the effect of ESS and MSS on primary and secondary outcomes, and divided the above data into continuous data and binary data. For continuous data, the mean differences [standard mean differences (SMD) or weighted mean difference (WMD)] and 95% CIs were estimated. For binary outcome data, odds ratios (ORs) and 95% CIs were estimated using the Mantel–Haenszel method [16]. What’s more, the Cochran *Q* test was used to evaluate heterogeneity [17]. Also, *I*^2^ testing was applied to evaluate the magnitude of the heterogeneity between studies. If *I*^2^ ≤ 50%, the fixed effects model was used for analysis, or the random effects model would be chosen [16, 18]. We used Stata (version 12.0 SE) for all statistical analysis.

## Result

### Study selection and characteristics

**Fig. 1:**
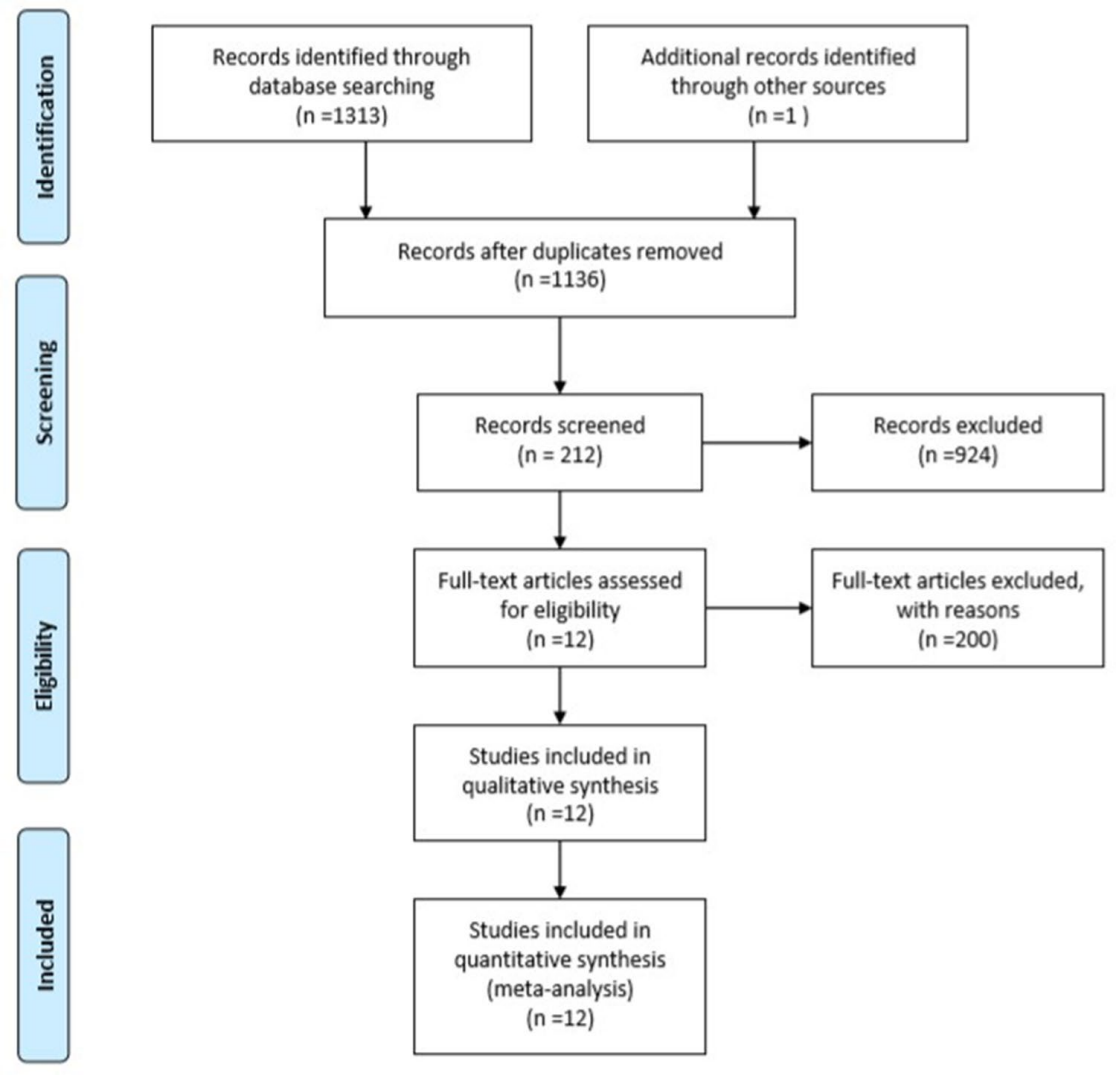
The flowchart shows the literature search and study selection process according to the PRISMA guidelines

Totally, 1314 articles were identified, of which 178 were excluded because of duplication. After evaluating the title and abstract, 924 articles were removed. The full-text of the remaining 212 articles was reviewed, and we also contacted some authors to ask for unpublished data. For the qualitative and quantitative analysis, only 12 studies eventually remained. It is in Fig. 1 that thoroughly shows the PRISMA diagram of the literature search and selection procedures. All selected studies, including 673 patients undergoing ESS or MSS, were written in English and published from 2014 to 2019. Two studies were randomized controlled trials (RCTs), and the others were non-randomized cohort studies (nRCSs). Patients who underwent ESS belonged to the exposure group, and the control group included all patients who underwent MSS. The detailed information of the 12 trials was partially exhibited in Table 1.

**Table 1:** Study characteristics

Lead author (year)	Country	Study design	Patients (no.)			Study population	Mean age (years)		Mean follow-up (month)		Outcomes
			Total	ESS*	MSS*		EDG	MCG	EDG	MCG	
Kojima et al. (2014) [12]	Japan	nRCS	56	15	41	Otosclerosis and congenital stapedial fixation	40.1	49.47	8.6	8.6	a, e
Daneshi et al. (2015)	Iran	nRCS	34	19	15	Otosclerosis	36.8	36.8	7.42	7.42	a, b, d, e
Iannella et al. (2016) [24]	Italy	nRCS	40	20	20	Otosclerosis	44.3	45.3	10.3	9	a, b, c, d, e
Sproat et al. (2017) [23]	Turkey	nRCS	81	34	47	Otosclerosis	47	51	5	10	a, c
Surmelioglu et al. (2017) [22]	UK	nRCS	46	22	24	Otosclerosis	39.2	44.3	15.8	21.5	a, b, c, d, e
Moneir et al. (2018) [21]	USA	nRCS	42	22	24	Otosclerosis	33.6	33	4.5	5.5	a, d, e
Ardic et al. (2018) [6]	India	nRCS	94	37	57	Otosclerosis	42.2	43.8	12	12	a, e
Bhardwaj et al. (2018) [8]	Egypt	RCT	40	20	20	Otosclerosis	33.1	32.9	12	12	a, b, c, d, e
Kuo et al. (2018) [7]	China Taipei	nRCS	30	17	13	Otosclerosis	49.6	54.2	N	N	a, b
Gulsen et al. (2019) [2]	Turkey	nRCS	72	38	34	Otosclerosis	32.6	35.6	13.2	12.1	a, b, c, d, e
Tolisano et al. (2019) [3]	USA	nRCS	74	22	52	Otosclerosis	10.5	10.5	7.1	27	a, b, c, d, e
Das et al. (2019) [20]	India	RCT	64	32	32	Otosclerosis and congenital stapedial fixation	40	38.5	1	1	a, d, e

^*^Items of evaluation: *ESS* endoscopic stapes surgery, *MSS* microscopic stapes surgery

^a^Postoperative hearing

^b^Scutum drilling

^c^Chorda tympani handling

^d^Operation time

^e^Postoperative complications

### Quality assessment

The MINORS Scale, developed for randomized surgical trials and observational studies in surgery by a group of surgeons, was applied to evaluating the methodological quality of included studies [19]. 12 items were included and the items are scored 0 (not reported), 1 (reported but inadequate) or 2 (reported and adequate). According to the aggregate scores of each study, we allocated different score ranges a “high” (0–8), “middle” (9–16) and “low” (17–24) risk of bias, because of the lack of explicit definition in the MINORS Scale. The exact scoring of individual studies was presented in Table 2. Ultimately, four studies had low risk of bias while eight studies had middle risk of bias. The main reasons of risk of bias, as the table exhibited, were the lack of consecutive patients, non-blind trails and the absence of prospective calculation of the study size. Fortunately, no studies of high risk of bias were included.

**Table 2:** Risk of bias analysis

Study	1*	2	3	4	5	6	7	8	9	10	11	12	Total
Kojima et al. (2014) [12]	2	0	0	1	0	2	1	0	2	0	1	1	10
Daneshi et al. (2015)	2	0	0	1	0	2	1	0	2	0	1	1	10
Iannella et al. (2016) [24]	2	0	0	1	0	2	1	0	2	2	1	1	12
Sproat et al. (2017) [23]	2	0	2	1	0	2	1	0	2	0	1	1	12
Surmelioglu et al. (2017) [22]	2	0	0	1	0	2	1	0	2	0	1	2	11
Moneir et al. (2018) [21]	2	0	2	1	0	2	1	0	2	0	1	2	15
Ardic et al. (2018) [6]	2	0	0	1	0	2	1	0	0	2	1	1	10
Bhardwaj et al. (2018) [8]	2	2	2	2	1	2	2	0	2	2	1	1	19
Kuo et al. (2018) [7]	2	1	1	2	0	0	1	0	2	0	1	2	12
Gulsen et al. (2019) [2]	2	2	2	2	1	2	1	0	2	1	1	2	18
Tolisano et al. (2019) [3]	2	2	1	2	0	2	1	0	2	2	1	2	17
Das et al. (2019) [20]	2	2	2	2	1	1	2	0	2	2	1	2	19

^*^Items of evaluation: 1 a clearly stated aim, 2 inclusion of consecutive patients, 3 prospective collection of data, 4 endpoints appropriate to the aim of the study, 5 unbiased assessment of the study endpoint, 6 follow-up period appropriate to the aim of the study, 7 loss to follow-up less than 5%, 8 prospective calculation of the study size, 9 an adequate control group, 10 contemporary groups, 11 baseline equivalence of groups, 12 adequate statistical analyses

### Primary outcomes

**Fig. 2:**
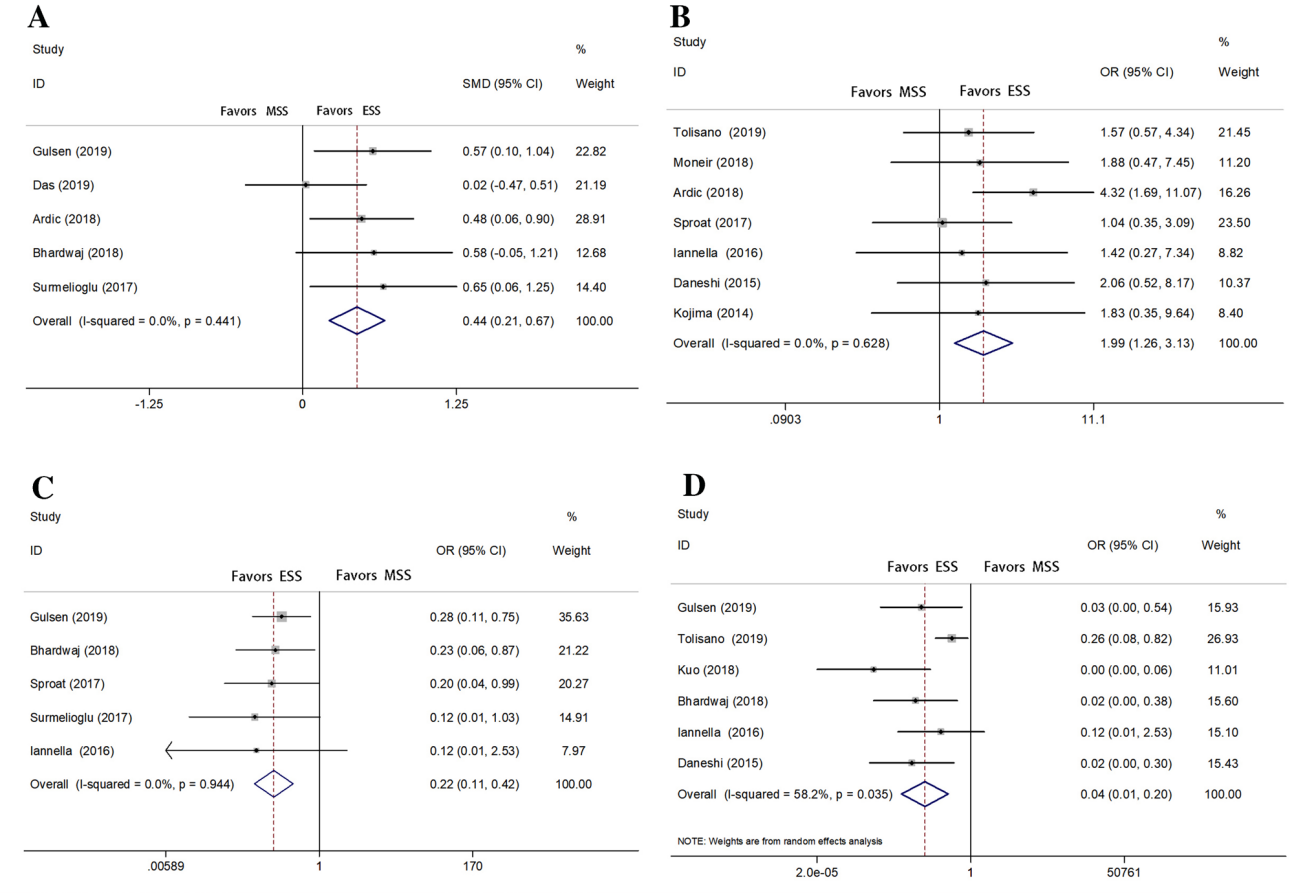
Forest plots with respect to primary outcomes (**a** average postoperative auditory gain **b** postoperative ABG of 10 dB or less **c** chorda tympani handling **d** bone curettage)

Eight studies [2, 3, 6–8, 20–22] recorded the outcome of average postoperative auditory gain (APAG), but only five articles [2, 6, 8, 20, 22] have reported both the value of mean and standard deviation. The standard mean difference (SMD) of APAG was 0.44 [95% CI 0.21–0.67; *P* = 0.441; heterogeneity, *I*^2^ = 0%] (Fig. 2a). Because of *I*^2^ ≤ 50%, we chose fixed effects models for analysis. Obviously, there was a statistically significant difference in favor of ESS. Seven studies [3, 6, 12, 13, 21, 23, 24] recorded the outcome of postoperative air–bone gap (ABG) of 10 dB or less. The OR was 1.99 [95% CI 1.26–3.13; *P* = 0.6.28; heterogeneity, *I*^2^ = 0%] (Fig. 2b), and fixed effects models were applied to analysis. Significant difference also existed statistically in favor of ESS. Besides, when excluding the outcome of Tolisano’s study [3] resulting from patients who were all children with narrow meatus, the statistically significant difference is obvious. The OR of pooling data, containing five studies [2, 8, 22–24], of chorda tympani handling was 0.22 [95% CI 0.11–0.42; *P* = 0.944; heterogeneity, *I*^2^ = 0%] (Fig. 2c). The *I*^2^ statistical analysis suggested mild heterogeneity, and the rate of chorda tympani handling is significantly higher with MSS. Similarly, the rate of bone curettage was reduced by 96% with endoscope. The OR, favoring ESS apparently, was 0.04 [95% CI 0.01–0.20; *P* = 0.035; heterogeneity, *I*^2^ = 58.2%] (Fig. 2d), and a random effects model was employed because of *I*^2^ ≥ 50% [2, 3, 7, 8, 13, 24].

### Secondary outcomes

**Fig. 3:**
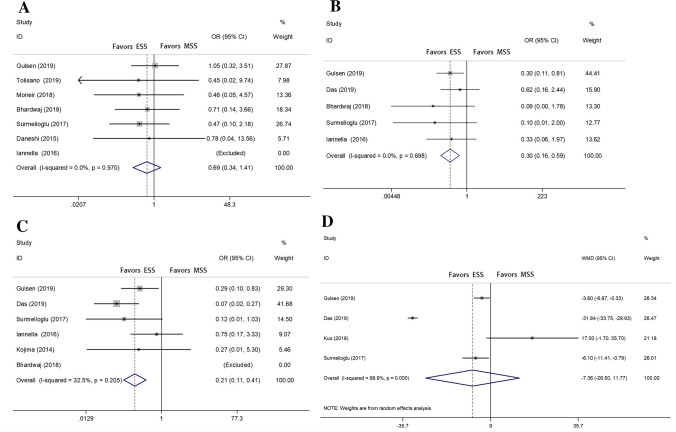
Forest plots with respect to secondary outcomes (**a** postoperative dizziness **b** postoperative pain **c** dysgeusia **d** average operative time)

The number of postoperative dizziness was recorded in seven studies [2, 3, 8, 13, 21, 22, 24], and Iannella’s study [24] was removed automatically by software because of the same outcomes in two groups. The OR was 0.69 [95% CI 0.34–1.41; *P* = 0.970; heterogeneity, *I*^2^ = 0%] in fixed effects models. There was no statistically significant difference between ESS and MSS (Fig. 3a). Besides, the outcomes of postoperative pain [2, 8, 20, 22, 24] and postoperative dysgeusia [2, 12, 20, 22, 24] were respectively recorded in five studies. The OR of pain was 0.30 [95% CI 0.16–0.59; *P* = 0.698; heterogeneity, *I*^2^ = 0%] (Fig. 3b) and the OR of dysgeusia was 0.21 [95% CI 0.11–0.41; *P* = 0.205; heterogeneity, *I*^2^ = 32.5%] (Fig. 3c). Both of them favored ESS with the statistically significant difference. Nine studies compared the average operative time [2, 3, 7, 8, 13, 20–22, 24], but only four studies were included resulting from the paucity of the standard deviation [2, 7, 20, 22]. No significant difference was showed between the two operative approaches [WMD = − 7.364; 95% CI − 26.50–11.77; *P* = 0.451; heterogeneity, *I*^2^ = 98.9%] (Fig. 3d). According to the value of *I*^2^, the heterogeneity is considerable. We did not perform any funnel plot, since less than ten studies were included in each analysis.

## Discussion

Undoubtedly, endoscopes, an promising technique, is superior to microscopic surgery for considerable benefits, such as improved surgical field and panoramic view of the complex middle ear structure, leading to fewer bone removal and, to some extent, less injury to CTN according to previous experience [5]. In this meta-analysis, we pooled the largest amount of record to date with direct comprehensive comparison of ESS and MSS since 2014 to 2019.

The situation of postoperative hearing is the most valuable item to measure whether the surgery is successful. To the best of our knowledge, the item, APAG, was firstly evaluated in our meta-analysis. As can be observed, pooled data based on five studies showed a significantly less value of postoperative ABG in ESS. On the other hand, closure of the ABG to < 10 dB is a widely reported outcome measure for success in stapes surgery [23]. Unlike previous two meta-analyses [4, 5], it is a statistical difference favoring the endoscope that exists in our study. The outcome of postoperative hearing obviously indicated that a superiority of ESS over MSS is indeed present.

Extent of chorda tympani manipulation, to be sure, is also an imperative, even incredibly difficult, point in stapes surgery [25–27]. As expected, our study has reached statistical significance in favor of the endoscope. Thanks to angled endoscopes, wider panoramic view of the surgical field, when accessed from inferior to superior direction from the hypotympanum, was provided in ESS [9, 11]. But in the microscopic group, it was ineluctable to manipulate CTN because of limited visualization. However, in light of all patients who were children with narrow external canal, we removed the results of Tolisano et al. [3], or no statistical difference would exist in our analysis.

As far as we know, alteration in taste sensation is directly associated with tympani manipulation during surgery [25, 28]. According to reports before, many patients who underwent MSS suffered from postoperative taste disturbance, which can be present in 20–60% of patients after this surgery [22, 28]. In contrast, some authors reported that endoscopic surgery, for avoiding handling the chorda tympani, may result in a reduced incidence of postoperative dysgeusia [24, 26]. In our analysis including six studies, we find statistical difference supporting the above conclusion similar to Nikolaos’s et al. outcome [4] but different from Koukkoullis’s et al. analysis [5].

Adequate exposure is critical both in ESS and in MSS. The visibility of the footplate area is crucial to ensure accurate insertion of the piston and minimal invasion to the fragile inner ear structures [8, 9, 29]. Yet, in MSS, the postero-superior bony canal wall often encumbers the view of the surgical field so that bony canal wall has to be removed for better visualization. Conversely, the incidence of extensive bone curettage or drilling was minimal in ESS, and wider panoramic view is obtained with fewer sacrifice of any soft tissue lying ahead [9]. What’s more, it was fewer patients, after endoscopic stapedotomy, that complained of postoperative pain [11, 20]. Indeed, our meta-analysis, in accordance with these recent findings, showed a statistically significant difference. On the contrary, a prior meta-analysis based on four retrospective studies which was published in 2018 showed, in the main, no differences in postoperative pain between the endoscopic and microscopic approaches, but we should not neglect that the most recent studies were not included [4]. Therefore, we have reasons to believe that our latest conclusion is more credible.

Postoperative dizziness was also a substantial complaint affecting the patients’ quality of life after surgery. Patients performed with ESS would suffer, theoretically speaking, less with postoperative dizziness because of, it is thought, reduced operation of the stapes footplate due to better visualization. Nevertheless, no significant difference was found in our analysis. In light of the above outcome, we postulate that the endoscope temperature probably could affect the inner ear leading to unknown influence in spite of the minimally invasive approach [30]. What’s more, postoperative dizziness, in our opinion, is mainly related to the trauma severity of fenestration and the length of the prosthesis rather than the type of surgical approach. Obviously, further studies are needed to ascertain our hypothesis.

Theoretically, the endoscopic operative field was clear and otologic surgeons could easily identify anatomy of the middle ear leading to sacrifice fewer bony structures or even not, which could, to some extent, shorten the operative time. Conversely, the operative time, in some studies [3, 7], is longer for endoscopic than microscopic surgeries whether surgeons are novice or experienced, implying this is not simply due to the “learning curve”. Admittedly, surgeons also spent additional time on continually wiping the fogged or blood-stained endoscope or on stopping bleeding with one-handed surgery. Therefore, there is a need for high-quality pooled data in the future to fully compare ESS with MSS on operative time.

Despite the aforementioned positive conclusions of endoscopic stapes surgery, the limitations of endoscopy, such as only one hand operation, the two-dimensional vision leading to lack of depth perception and potentially long learning curve, must be weighed in the determination of introducing endoscopic technology in stapes surgery [9, 11, 30]. To get rid of above limitations, the use of a robotic system to hold endoscope, and the application of 3D endoscopes to provide stereoscopic vision was recommended [10, 31]. But those equipment are too immature to be applied. As a matter of fact, with the experience of surgery increasing gradually, the otologic doctors are accustomed to working with the two-dimensional vision, so that the surgical manipulation will be no longer limited by the lack of stereoscopic vision [8, 24]. On the other hand, the patient’s postoperative health and, of course, quality of life, like taste disorder, pain and the requirement of minimally invasive surgery, are supposed to be considered and ESS seems to be superior to MSS in those areas.

There are, to be sure, still some limitations in this study. First, a limitation of this analysis is that only two RCTs were included while others were nRCSs, which is almost usually regarded as a relatively low level of evidence. Therefore, it is, in a way, nearly impossible to eliminated potential bias completely. For example, surgical procedures, among all of involved studies, were not standardized, and surgery in some studies was even performed by different surgeons. This bias may result in an underestimation of the efficacy of the experimental procedure. What's more, children who were patients with congenital stapedial fixation were also included in our study. This study population, undoubtedly, would result in a falsely decreased efficacy, since these cases, generally speaking, are related to poor postoperative hearing outcomes and a higher incidence of complications. Additionally, further bias is the size of prosthesis. According to a previous meta-analysis, Laske et al. found it more likely for 0.6 mm pistons than for 0.4 mm pistons to gain an air–bone gap closure of less than 10 dB [29]. The discrepancy in the size of prosthesis might, to some extent, affect the outcomes of postoperative hearing. Besides, APAG can be influenced by the preoperative air–bone gap leading to potential bias. Additionally, endoscopic groups had relatively shorter follow-up than microscopic groups as this new technique was just adopted for a while. Despite these limitations, the sensitivity analyses and the low heterogeneity observed, as seen here, supported the robustness and consistency of our results. The deduction, to put it differently, of our study is valuable.

## Conclusion

In spite of the fact that, admittedly, some of the including studies had small sample sizes and relatively poor methodological quality, a consistently superior effect of the ESS group, in terms of total effectiveness, was still showed in the analysis of the pooled data, when compared to the MSS group. Although further studies are needed, our findings clearly support the use of endoscopy in stapes surgery. The future of ESS, we believe, is blazing bright.
